# *catena*-Poly[[trimethyl­tin(IV)]-μ-2,4,6-trichloro­benzoato]

**DOI:** 10.1107/S1600536808040798

**Published:** 2008-12-10

**Authors:** Liyuan Wen, Handong Yin, Wenkuan Li, Daqi Wang

**Affiliations:** a College of Chemistry and Chemical Engineering, Liaocheng University, Shandong 252059, People’s Republic of China

## Abstract

In the title compound, [Sn(CH_3_)_3_(C_7_H_2_Cl_3_O_2_)]_*n*_, the tin(IV) atom exhibits a slightly distorted trigonal-bipyramidal geometry with two O atoms of symmetry-related carboxyl­ate groups in the axial positions and three methyl groups in the equatorial positions. In the crystal structure, the metal atoms are linked by carboxyl­ate bridges into polymeric chains extending along the *b* axis.

## Related literature

For related structures, see: Wang *et al.* (2007 ▶); Ma *et al.* (2006 ▶).
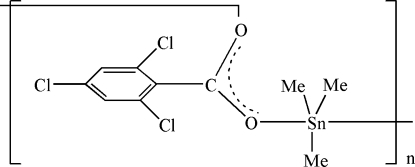


## Experimental

### 

#### Crystal data

[Sn(CH_3_)_3_(C_7_H_2_Cl_3_O_2_)]
*M* *_r_* = 388.25
Monoclinic, 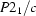

*a* = 9.8457 (10) Å
*b* = 9.6891 (9) Å
*c* = 15.3028 (19) Å
β = 106.761 (1)°
*V* = 1397.8 (3) Å^3^
*Z* = 4
Mo *K*α radiation
μ = 2.38 mm^−1^
*T* = 298 (2) K
0.42 × 0.18 × 0.08 mm

#### Data collection

Bruker SMART CCD area-detector diffractometer
Absorption correction: multi-scan (*SADABS*; Sheldrick, 1996 ▶) *T*_min_ = 0.434, *T*_max_ = 0.832
6983 measured reflections
2469 independent reflections
1996 reflections with *I* > 2σ(*I*)
*R*_int_ = 0.025

#### Refinement

*R*[*F* ^2^ > 2σ(*F* ^2^)] = 0.028
*wR*(*F* ^2^) = 0.085
*S* = 1.01
2469 reflections
145 parameters
H-atom parameters constrained
Δρ_max_ = 0.68 e Å^−3^
Δρ_min_ = −0.37 e Å^−3^

### 

Data collection: *SMART* (Siemens, 1996 ▶); cell refinement: *SAINT* (Siemens, 1996 ▶); data reduction: *SAINT*; program(s) used to solve structure: *SHELXS97* (Sheldrick, 2008 ▶); program(s) used to refine structure: *SHELXL97* (Sheldrick, 2008 ▶); molecular graphics: *SHELXTL* (Sheldrick, 2008 ▶); software used to prepare material for publication: *SHELXTL*.

## Supplementary Material

Crystal structure: contains datablocks I, global. DOI: 10.1107/S1600536808040798/rz2273sup1.cif

Structure factors: contains datablocks I. DOI: 10.1107/S1600536808040798/rz2273Isup2.hkl

Additional supplementary materials:  crystallographic information; 3D view; checkCIF report

## Figures and Tables

**Table 1:** Selected bond lengths (Å)

Sn1—C9	2.107 (5)
Sn1—C10	2.116 (5)
Sn1—C8	2.123 (4)
Sn1—O1	2.212 (3)
Sn1—O2^i^	2.467 (3)

Symmetry code: (i) 
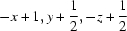
.

**Fig. 1.**
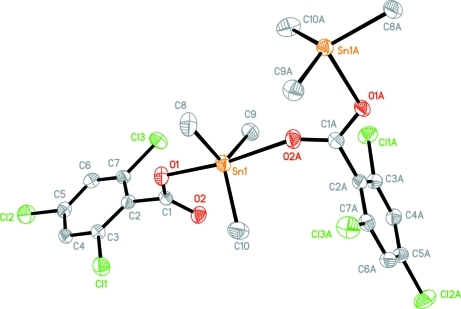
The molecular structure of the compound, showing 50% probability displacement ellipsoids. H atoms are omitted for clarity. Symmetry code: (A) = -x + 1,y + 1/2,-z + 1/2.

**Fig. 2.**
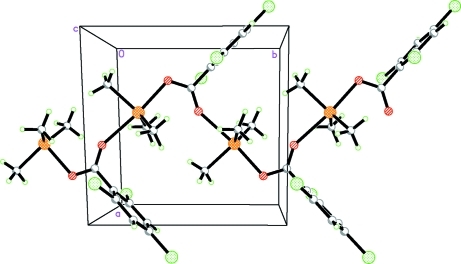
View of the one-dimensional chain structure extending along the b axis.

## supplementary crystallographic information

### Comment

Organoantin(IV) derivatives have recently attracted considerable attention due to the significant antimicrobial properties (Wang *et al.*, 2007). As a part of our ongoing investigations in this field, we have synthesized the title compound and present its crystal structure here.

In the title compound (Fig. 1), the Sn—O bond distances  (Table 1) are comparable to those found in related organotin carboxylates (Ma *et al.*, 2006). The Sn atom assumes a slightly distorted trigonal-bipyramidal coordination geometry, provided by two O atoms of symmetry related carboxylate groups at the axial positions and three methyl groups at the equatorial positions. In the crystal structure, the metal atoms are linked by carboxylate bridges into polymeric chains extending along the *b* axis (Fig. 2).

### Experimental

The reaction was carried out under nitrogen atmosphere. 2,4,6-Trichlorobenzoic acid (1 mmol) and sodium ethoxide (1.2 mmol) were added to a stirred solution of benzene (30 ml) in a Schlenk flask and stirred for 0.5 h. Trimethyltin chloride (1 mmol) was then added and the reaction mixture was stirred for 12 h at room temperature. The resulting clear solution was evaporated under vacuum. Colourless crystals suitable for X-ray analysis were obtained by slow evaporation of a dichloromethane/methanol (1:1 v/v) solution (yield 83%. m. p. 403K). Anal. Calcd (%) for C_10_H_11_Cl_3_O_2_Sn: C, 30.94; H, 2.86; O, 8.24; Sn, 30.58; Found (%): C, 30.89; H, 2.90; O, 8.31; Sn, 30.62.

### Refinement

H atoms were positioned geometrically, with methyl C—H distances of 0.96 Å and aromatic C—H distances of 0.93 Å, and refined as riding on their parent atoms, with *U*_iso_(H) = 1.2 *U*_eq_(C) or 1.5 *U*_eq_(C) for the methyl groups.

### Crystal data

**Table:**

[Sn(CH_3_)_3_(C_7_H_2_Cl_3_O_2_)]	*F*(000) = 752
*M**_r_* = 388.25	*D*_x_ = 1.845 Mg m^−^^3^
Monoclinic, *P*2_1_/*c*	Mo *K*α radiation, λ = 0.71073 Å
Hall symbol: -P 2ybc	Cell parameters from 3337 reflections
*a* = 9.8457 (10) Å	θ = 2.5–27.6°
*b* = 9.6891 (9) Å	µ = 2.38 mm^−^^1^
*c* = 15.3028 (19) Å	*T* = 298 K
β = 106.761 (1)°	Block, colourless
*V* = 1397.8 (3) Å^3^	0.42 × 0.18 × 0.08 mm
*Z* = 4	

### Data collection

**Table:**

Bruker SMART CCD area-detector diffractometer	2469 independent reflections
Radiation source: fine-focus sealed tube	1996 reflections with *I* > 2σ(*I*)
graphite	*R*_int_ = 0.025
φ and ω scans	θ_max_ = 25.0°, θ_min_ = 2.2°
Absorption correction: multi-scan (*SADABS*; Sheldrick, 1996)	*h* = −9→11
*T*_min_ = 0.434, *T*_max_ = 0.832	*k* = −10→11
6983 measured reflections	*l* = −18→15

### Refinement

**Table:**

Refinement on *F*^2^	Primary atom site location: structure-invariant direct methods
Least-squares matrix: full	Secondary atom site location: difference Fourier map
*R*[*F*^2^ > 2σ(*F*^2^)] = 0.028	Hydrogen site location: inferred from neighbouring sites
*wR*(*F*^2^) = 0.085	H-atom parameters constrained
*S* = 1.01	*w* = 1/[σ^2^(*F*_o_^2^) + (0.048*P*)^2^ + 1.1107*P*] where *P* = (*F*_o_^2^ + 2*F*_c_^2^)/3
2469 reflections	(Δ/σ)_max_ < 0.001
145 parameters	Δρ_max_ = 0.68 e Å^−^^3^
0 restraints	Δρ_min_ = −0.37 e Å^−^^3^

### Fractional atomic coordinates and isotropic or equivalent isotropic displacement parameters (Å^2^)

**Table:**

	*x*	*y*	*z*	*U*_iso_*/*U*_eq_	
Sn1	0.41662 (3)	0.26723 (3)	0.24850 (2)	0.03954 (13)	
Cl1	0.13395 (13)	−0.14643 (14)	0.30795 (8)	0.0553 (3)	
Cl2	−0.17245 (14)	−0.41671 (14)	0.00986 (9)	0.0685 (4)	
Cl3	0.21742 (14)	−0.04603 (14)	−0.01909 (8)	0.0589 (3)	
O1	0.2441 (3)	0.1152 (3)	0.1981 (2)	0.0436 (7)	
O2	0.4091 (3)	−0.0438 (3)	0.2076 (2)	0.0483 (8)	
C1	0.2834 (4)	−0.0058 (4)	0.1860 (3)	0.0368 (9)	
C2	0.1682 (4)	−0.1073 (4)	0.1414 (3)	0.0357 (9)	
C3	0.0947 (4)	−0.1793 (4)	0.1922 (3)	0.0379 (9)	
C4	−0.0092 (5)	−0.2761 (4)	0.1529 (3)	0.0416 (10)	
H4	−0.0557	−0.3248	0.1881	0.050*	
C5	−0.0410 (5)	−0.2974 (4)	0.0609 (3)	0.0429 (11)	
C6	0.0256 (5)	−0.2269 (4)	0.0067 (3)	0.0449 (11)	
H6	0.0004	−0.2414	−0.0560	0.054*	
C7	0.1307 (4)	−0.1341 (4)	0.0483 (3)	0.0396 (10)	
C8	0.2673 (5)	0.4245 (5)	0.2502 (4)	0.0582 (13)	
H8A	0.3162	0.5044	0.2809	0.087*	
H8B	0.2023	0.3917	0.2818	0.087*	
H8C	0.2158	0.4484	0.1887	0.087*	
C9	0.4924 (6)	0.2466 (5)	0.1337 (3)	0.0510 (12)	
H9A	0.5867	0.2092	0.1523	0.077*	
H9B	0.4938	0.3355	0.1062	0.077*	
H9C	0.4312	0.1857	0.0902	0.077*	
C10	0.5129 (6)	0.1758 (6)	0.3770 (3)	0.0617 (14)	
H10A	0.6022	0.1359	0.3770	0.093*	
H10B	0.4520	0.1051	0.3886	0.093*	
H10C	0.5283	0.2449	0.4238	0.093*	

### Atomic displacement parameters (Å^2^)

**Table:**

	*U*^11^	*U*^22^	*U*^33^	*U*^12^	*U*^13^	*U*^23^
Sn1	0.03773 (19)	0.0345 (2)	0.0475 (2)	−0.00090 (12)	0.01410 (14)	−0.00256 (13)
Cl1	0.0537 (7)	0.0745 (9)	0.0398 (7)	−0.0114 (6)	0.0167 (5)	−0.0052 (6)
Cl2	0.0641 (8)	0.0599 (8)	0.0692 (9)	−0.0214 (7)	−0.0003 (6)	−0.0076 (7)
Cl3	0.0717 (8)	0.0616 (8)	0.0511 (7)	−0.0064 (6)	0.0300 (6)	0.0062 (6)
O1	0.0357 (15)	0.0308 (16)	0.066 (2)	−0.0010 (12)	0.0176 (14)	−0.0100 (14)
O2	0.0367 (17)	0.0375 (17)	0.068 (2)	0.0041 (13)	0.0106 (14)	0.0015 (15)
C1	0.038 (2)	0.034 (2)	0.040 (2)	−0.0021 (18)	0.0155 (19)	0.0017 (18)
C2	0.035 (2)	0.028 (2)	0.042 (2)	0.0022 (17)	0.0089 (18)	−0.0017 (18)
C3	0.040 (2)	0.037 (2)	0.038 (2)	−0.0023 (19)	0.0133 (19)	−0.0009 (19)
C4	0.038 (2)	0.036 (2)	0.051 (3)	−0.0047 (18)	0.012 (2)	0.003 (2)
C5	0.039 (2)	0.033 (2)	0.051 (3)	−0.0032 (19)	0.005 (2)	−0.007 (2)
C6	0.049 (3)	0.045 (3)	0.038 (3)	0.004 (2)	0.009 (2)	−0.005 (2)
C7	0.041 (2)	0.035 (2)	0.045 (3)	0.0037 (18)	0.0144 (19)	0.0031 (19)
C8	0.045 (3)	0.045 (3)	0.086 (4)	−0.004 (2)	0.022 (3)	−0.019 (3)
C9	0.055 (3)	0.052 (3)	0.051 (3)	−0.002 (2)	0.023 (2)	0.001 (2)
C10	0.078 (4)	0.056 (3)	0.047 (3)	−0.014 (3)	0.011 (3)	0.004 (2)

### Geometric parameters (Å, °)

**Table:**

Sn1—C9	2.107 (5)	C4—C5	1.366 (6)
Sn1—C10	2.116 (5)	C4—H4	0.9300
Sn1—C8	2.123 (4)	C5—C6	1.377 (7)
Sn1—O1	2.212 (3)	C6—C7	1.380 (6)
Sn1—O2^i^	2.467 (3)	C6—H6	0.9300
Cl1—C3	1.730 (4)	C8—H8A	0.9600
Cl2—C5	1.744 (4)	C8—H8B	0.9600
Cl3—C7	1.740 (4)	C8—H8C	0.9600
O1—C1	1.265 (5)	C9—H9A	0.9600
O2—C1	1.241 (5)	C9—H9B	0.9600
O2—Sn1^ii^	2.467 (3)	C9—H9C	0.9600
C1—C2	1.508 (5)	C10—H10A	0.9600
C2—C7	1.390 (6)	C10—H10B	0.9600
C2—C3	1.392 (6)	C10—H10C	0.9600
C3—C4	1.390 (6)		
			
C9—Sn1—C10	124.3 (2)	C6—C5—Cl2	118.7 (4)
C9—Sn1—C8	119.4 (2)	C5—C6—C7	118.0 (4)
C10—Sn1—C8	114.6 (2)	C5—C6—H6	121.0
C9—Sn1—O1	93.83 (16)	C7—C6—H6	121.0
C10—Sn1—O1	97.96 (16)	C6—C7—C2	122.4 (4)
C8—Sn1—O1	91.00 (15)	C6—C7—Cl3	118.5 (4)
C9—Sn1—O2^i^	84.92 (15)	C2—C7—Cl3	119.1 (3)
C10—Sn1—O2^i^	88.16 (16)	Sn1—C8—H8A	109.5
C8—Sn1—O2^i^	83.90 (15)	Sn1—C8—H8B	109.5
O1—Sn1—O2^i^	173.28 (10)	H8A—C8—H8B	109.5
C1—O1—Sn1	115.6 (2)	Sn1—C8—H8C	109.5
C1—O2—Sn1^ii^	148.7 (3)	H8A—C8—H8C	109.5
O2—C1—O1	124.0 (4)	H8B—C8—H8C	109.5
O2—C1—C2	119.3 (4)	Sn1—C9—H9A	109.5
O1—C1—C2	116.6 (3)	Sn1—C9—H9B	109.5
C7—C2—C3	116.9 (4)	H9A—C9—H9B	109.5
C7—C2—C1	121.9 (4)	Sn1—C9—H9C	109.5
C3—C2—C1	121.2 (4)	H9A—C9—H9C	109.5
C4—C3—C2	122.1 (4)	H9B—C9—H9C	109.5
C4—C3—Cl1	119.1 (3)	Sn1—C10—H10A	109.5
C2—C3—Cl1	118.7 (3)	Sn1—C10—H10B	109.5
C5—C4—C3	118.0 (4)	H10A—C10—H10B	109.5
C5—C4—H4	121.0	Sn1—C10—H10C	109.5
C3—C4—H4	121.0	H10A—C10—H10C	109.5
C4—C5—C6	122.6 (4)	H10B—C10—H10C	109.5
C4—C5—Cl2	118.8 (4)		
			
C9—Sn1—O1—C1	61.1 (3)	C1—C2—C3—Cl1	2.0 (5)
C10—Sn1—O1—C1	−64.4 (3)	C2—C3—C4—C5	−1.5 (7)
C8—Sn1—O1—C1	−179.3 (3)	Cl1—C3—C4—C5	178.2 (3)
Sn1^ii^—O2—C1—O1	154.5 (4)	C3—C4—C5—C6	−0.1 (7)
Sn1^ii^—O2—C1—C2	−26.3 (8)	C3—C4—C5—Cl2	−179.2 (3)
Sn1—O1—C1—O2	6.1 (5)	C4—C5—C6—C7	1.7 (7)
Sn1—O1—C1—C2	−173.0 (3)	Cl2—C5—C6—C7	−179.2 (3)
O2—C1—C2—C7	−82.9 (5)	C5—C6—C7—C2	−1.8 (7)
O1—C1—C2—C7	96.3 (5)	C5—C6—C7—Cl3	179.1 (3)
O2—C1—C2—C3	96.8 (5)	C3—C2—C7—C6	0.2 (6)
O1—C1—C2—C3	−84.0 (5)	C1—C2—C7—C6	179.9 (4)
C7—C2—C3—C4	1.5 (6)	C3—C2—C7—Cl3	179.3 (3)
C1—C2—C3—C4	−178.2 (4)	C1—C2—C7—Cl3	−1.0 (5)
C7—C2—C3—Cl1	−178.3 (3)		

Symmetry codes: (i) −*x*+1, *y*+1/2, −*z*+1/2; (ii) −*x*+1, *y*−1/2, −*z*+1/2.
